# Massive hematuria due to a congenital renal arteriovenous malformation mimicking a renal pelvis tumor: a case report

**DOI:** 10.1186/1752-1947-2-144

**Published:** 2008-05-05

**Authors:** P Sountoulides, I Zachos, K Paschalidis, I Asouhidou, A Fotiadou, A Bantis, M Palasopoulou, T Podimatas

**Affiliations:** 1 Urology Department, "Agios Andreas" Hospital of Patras, Greece; 2 Urology Department, General Hospital of Veria, 59100, Greece; 3 Anesthesiology Department, "Papageorgiou" Hospital, Thessaloniki, Greece; 4 Department of Interventional Radiology, "Papageorgiou" Hospital, Thessaloniki, Greece; 5 Medical School, University of Alexandroupolis, Dragana 68100, Alexandroupolis, Greece

## Abstract

**Introduction:**

Congenital renal arteriovenous malformations (AVMs) are very rare benign lesions. They are more common in women and rarely manifest in elderly people. In some cases they present with massive hematuria. Contemporary treatment consists of transcatheter selective arterial embolization which leads to resolution of the hematuria whilst preserving renal parenchyma.

**Case presentation:**

A 72-year-old man, who was heavy smoker, presented with massive hematuria and flank pain. CT scan revealed a filling defect caused by a soft tissue mass in the renal pelvis, which initially led to the suspicion of a transitional cell carcinoma (TCC) of the upper tract, in view of the patient's age and smoking habits. However a subsequent retrograde study could not depict any filling defect in the renal pelvis. Selective right renal arteriography confirmed the presence of a renal AVM by demonstrating abnormal arterial communication with a vein with early visualization of the venous system. At the same time successful selective transcatheter embolization of the lesion was performed.

**Conclusion:**

This case highlights the importance of careful diagnostic work-up in the evaluation of upper tract hematuria. In the case presented, a congenital renal AVM proved to be the cause of massive upper tract hematuria and flank pain in spite of the initial evidence indicating the likely diagnosis of a renal pelvis tumor.

## Introduction

Renal arteriovenous malformations (AVMs) are rare lesions, and may be acquired or congenital. Acquired renal AVMs, otherwise called arteriovenous fistulae, represent about 70% of all AVMs and usually result from trauma, inflammation or percutaneous procedures involving the kidney (e.g. renal biopsy). Although rare, congenital renal AVMs can result in significant hematuria which may require arterial embolisation or open surgery. There are only a few case series in the literature describing the outcome of congenital arteriovenous malformations. We report a challenging case of a renal arteriovenous malformation in an elderly man presenting with severe hematuria.

## Case presentation

A 72-year-old man was admitted with right flank pain and massive hematuria with clot retention. The patient was a heavy smoker, and did not report any history of trauma, recent medical intervention or known lithiasis. He denied any bleeding disorder and was not taking any medications. His blood pressure was normal and so were his blood count, biochemical and coagulation parameters. A rinsing catheter was introduced and the hematuria resolved within a few days. Ultrasonographic examination of the kidneys and bladder was unremarkable.

**Figure 1:**
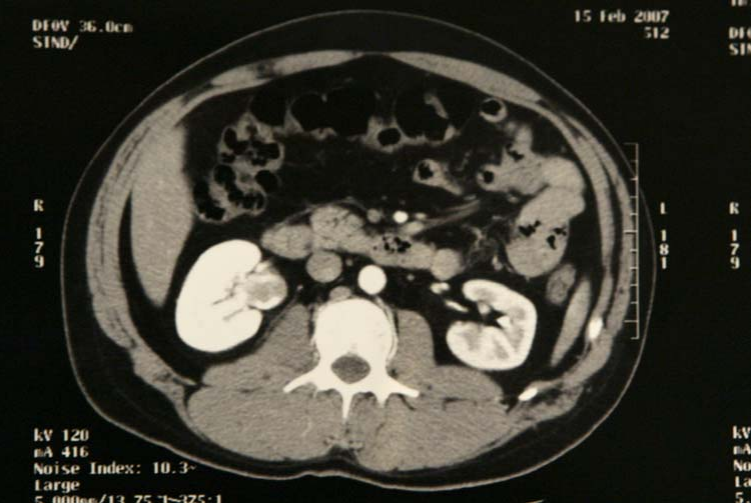
Axial computed tomography scan after intravenous administration of iodinated contrast material, arterial phase. A filling defect of soft tissue density is demonstrated within the right renal pelvis, which is also dilated. Differential diagnosis includes blood clot and tumor.

On a subsequent CT scan, a small soft tissue mass was depicted within the right renal pelvis. The lesion did not significantly enhance after injection of contrast medium and there was no dilatation of the affected renal unit. There was no evidence of urinary tract lithiasis or other pathology (Figure 1).

The CT scan could not differentiate between a blood clot and a tumor. In the presence of a filling defect in the renal pelvis, although slightly enhancing, the presence of an urothelial lesion had to be excluded. Urine cytology was negative. A cystoscopy with advancement of a ureteral catheter into the right pelvis was carried out in order to selectively obtain a sample for urine cytology and perform a retrograde study. Cystoscopy and upper tract cytology were both negative for a high-grade bladder tumor. The retrograde study of the right ureter and pelvicaliceal system did not demonstrate any filling defect in the right renal pelvis and calyces. Based on these findings, the CT findings were attributed to a blood clot in the right renal pelvis and the investigation proceeded with renal arteriography.

**Figure 2:**
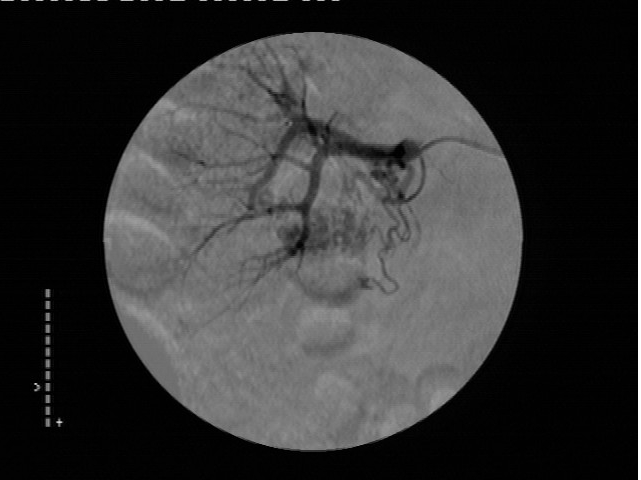
Selective digital subtraction arteriography of the right kidney showing an area of tortuous vascular channels located in the lower renal pole. The image taken a few seconds after the injection of contrast material demonstrates also early filling of the renal vein.

**Figure 3:**
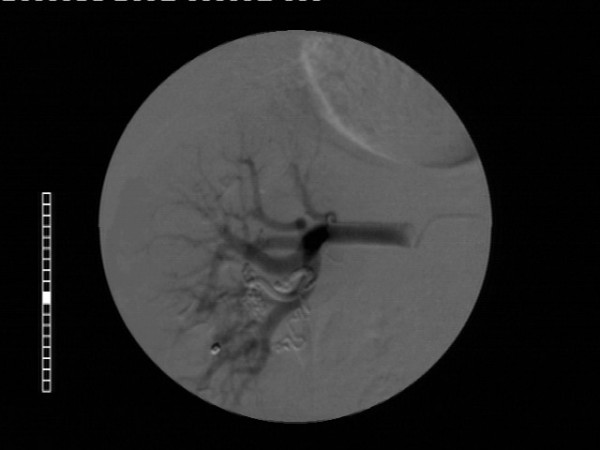
Image after transcatheter superselective embolization of the lesion with the use of coils. No vascular supply of the lesion is demonstrated.

Selective right renal arteriography was carried out shortly following the resolution of the hematuria, and demonstrated an area of tortuous, coiled vascular channels with early filling of the renal vein within two seconds after the start of the injection(Figure 2). Therefore, a right peripelvic renal AVM was diagnosed and a transcatheter superselective embolization of the lesion with the use of coils was performed successfully during the same session (Figure 3).

At follow-up one year later, with non-enhanced and enhanced CT, there were no abnormalities found. The patient remains free of symptoms.

## Discussion

Congenital renal arteriovenous malformations are considered to represent focal spontaneous failures of vascular development occurring between the 4^th ^and 10^th ^weeks of life [1]. However they usually remain asymptomatic until the 3^rd ^or 4^th ^decade of life.

Women are affected three times as often as men, and the right kidney is involved slightly more often than the left. Renal AVMs are rare causes of hematuria. Congenital renal AVMs are of two general types: cirsoid, with multiple varix-like vascular communications which represents a truly congenital form of arteriovenous malformation [2]; and aneurysmal, which is considered to be idiopathic, presents at a later age, and usually develops when a pre-existing arterial aneurysm erodes into an adjacent vein [3].

Hematuria that can be so severe as to be life-threatening is more characteristic of the congenital form of AVMs, presenting as the primary symptom in 3 out of 4 patients [3]. Hematuria is thought to result from an increase in renal venous pressure causing minute rupture of these thin-walled veins into the collecting system. This is why peripherally located AVMs however small they are, can cause massive hematuria.

In this case, a 72 year-old man presented with an episode of right renal colic and hematuria. Usual bleeding sources from the upper urinary tract at this age are tumours and stones, with AVMs being very rare.

Unenhanced helical CT can detect urinary stones with an accuracy of almost 97% [4]. In this patient no renal or ureteral stone was found. However the finding of a small soft tissue mass in the renal pelvis, combined with the patient's smoking history, made the diagnosis of a transitional cell carcinoma (TCC) of the upper tract most probable.

Surprisingly, the retrograde urography that followed showed the absence of a filling defect in the right pelvis. This, combined with the negative urine cytology from the affected side, was evidence that the finding on CT was probably a blood clot.

In view of these findings, some form of arterial malformation was considered as a possible cause of the hematuria, despite the advanced age of the patient. The diagnostic workout could proceed with Color Doppler Ultrasonography which is less invasive than arteriography and could theoretically have been of help in our case. However this study was not performed because of the strong evidence in favor of a renal AVM and the radiologist's relative lack of experience with this method of investigation.

Moreover in cases of suspected renal AVMs, selective renal arteriography and digital subtraction angiography can be both diagnostic and therapeutic, as in our case. Congenital AVMs exhibit unique arteriographic patterns, demonstrating small cirsoid tangles of vessels with multiple varix-like communications between the feeding artery and vein. Arteriovenous shunting is also observed, with early visualization of the draining vein [3], as in the case presented.

Transcatheter arterial embolization (TAE) has become the treatment option of choice for the management of severe hematuria caused by renal AVMs, even in cases of AVMs complicating pregnancies [5,6]. TAE has replaced open surgery for AVMs and can sometimes obviate the need for general anesthesia. With the evolution of techniques, it is now possible to perform highly selective embolisation with maximum preservation of the renal parenchyma.

Contemporary embolization techniques aim at permanently occluding the nidus of an AVM, in other words the multiple small connections between arteries and veins through which the blood shunts. Embolic agents that had been used in the past, such as resorbable gelfoam sponge, stainless steel or platinum microcoils, or polyvinyl alcohol, have been replaced mainly due to the high rate of recanalization of the AVMs and recurrence of hematuria with these methods and partly because of the potential risk of pulmonary embolism [7].

Absolute alcohol [7] or n-butyl 2-cyanoacrylate (NBCA) diluted in Lipiodol [8] seems to provide better outcomes in terms of safety, efficacy and duration of results.

TAE is generally considered a safe option for the treatment of AVMs, with the most significant complication being the reflux of agents into non-target areas resulting in a larger area of infarction [7]. The risk of post embolization syndrome (PES), characterized by fever, loin pain, nausea and vomiting, and that of pulmonary embolism have substantially decreased. Selective embolization of renal AVMs allows preservation of the renal parenchyma and therefore leads to minimal post embolization syndrome (PES) [9].

Congenital arteriovenous malformations of the kidney are rare causes of severe hematuria especially in young patients. However, in cases like the one presented here, symptoms, history and imaging studies may be misleading. A renal AVM was found to be the cause of massive hematuria in this 72-year-old man with a long-standing history of smoking; reminding us once again that in medicine often nothing is as obvious as it seems to be.

## Competing interests

The authors declare that they have no competing interests.

## Authors' contributions

PS was the one who prepared and edited the discussion section. PS and IZ and AB were the treating urologists, involved in the diagnostic work-up and management of the patient. IZ also performed the retrograde study and wrote the case presentation section. KP is the head of the Department of Urology and was also responsible for the format and revisions of the manuscript. IA and MP were the treating anaesthesiologists while TP and AF were the interventional radiologists who performed the embolization. TP and AF prepared the CT images and the relevant legends. All the authors have read and approved the final version of the manuscript.

## Consent

Written informed consent was obtained from the patient for publication of this Case report and any accompanying images. A copy of the written consent is available for review by the Editor-in-Chief of this journal.
